# Explaining the link among self-controlling and children parenting techniques and mental insurance of high school pupils

**Published:** 2015

**Authors:** E Sekhavati, M Rahimian Boogar, M Khodadost, R Afkari, Raoufi Atefeh

**Affiliations:** * Larestan School of Medical Sciences, Larestan, Iran,; ** Department of Epidemiology, School of Public Health, Shahid Beheshti University of Medical Sciences, Tehran, Iran,; *** Department of Microbiology, Jahrom Branch, Young Researcher Club, Islamic Azad University, Jahrom, Iran

**Keywords:** self-controlling, kid parenting technique, mental health

## Abstract

**Introduction:** One of the wellness estimation axes of various communities are the mental well-being of the communities. Health means an attempt to Self-actualization and development that exhibit in the adaptation of person's skills and experiences. No doubt mental health plays a major role in assuring efficiency in any organization and can be affected via different parameters. Accordingly, the current research conducted by the purpose of illustrating the relationship among self-managing and kid parenting techniques and mental health amongst high school pupils of Abadeh Town.

**Methodology:** In this sectional-correlation research, 375 pupils are taken and involved in the research in stage group sampling technique of Abadeh high schools. Tangney’s self-managing survey, Barry's kid parenting rate, Reef’s mental survey and a framework of demographic data utilized to obtain data. Information investigated utilizing software SPSS 19 or Pearson’s relationship coefficient analysis and stepwise multivariable regression investigation.

**Findings:** Information investigation depicts self-managing parameter has a great and opposite predictability regarding mental health parameter (t =0.003, = β-0.158, P=2.99). Therefore self-managing has great and opposite predictability regarding 2 parts of mental health rate i.e. self-approval (P= 0.0001, t=4.87, β= - 0.181) and dominance on conditions (P= 0.0001, t=3.807, β= - 0.200). The decisions represent the proximity of a consequence relationship among predictability of kid parenting techniques regarding mental health (p=0.01, F=3.85, r2= 0.031, r=0.177). These sequences reveal great predictability of kid parenting styles in 2 various ways in 2 methods of grinding (P=0.035, t= 2.12, β=0.113) and standard (P=0.014, t=2.437, β= 0.434). The Severe method has a reversed important connection in maximum features of mental health. Furthermore, they note that "authoritative method" parameter just has prediction capacity 0.143 based on mental health variance parameter. Plus combining 2 other parameters i.e. self-managing and cruel way, this value rises to 0.188 and 0.225. The greatest rate for prognosticating skill refers to the standard method of kid parenting straight and after that to self-managing and severe method reversely.

**Conclusion:** based on the significant relationship of kid parenting styles and self-managing in foretelling mental health, the need of notice to these parts is felt in describing the mental health of pupils as many as feasible. Therefore, it is suggested that education of kid parenting techniques is examined as a defensive and serving method for mental health in mental wellness plans for all teens particularly scholars therefore that parents could be satisfied in supporting their kids' emotional health and stopping their mental troubles via data and utilizing peculiar kid parenting techniques and withdrawing ineffective systems of kid parenting (as severe behavior).

## Introduction

In modern world, health views have received vaster perspectives and non-medical determining factors of health have been faced with special attention. Each of these determinants has influence on health status in their self or by influencing each other and causes injustice in health status. These determinants include genetics; way of life, environment, and psychic-social-economic status, etc. that have significant effect on health and its consequences like life quality and mental health. Psychic health is among things which humans search in their lives. Since psychic health has been considered vital need for improving humans’ quality of life, World Health Organization (WHO) describes health as a mood of welfare in which a person recognizes his capabilities, uses them in effective and productive way and is useful for his own society [**57**]. Psychic health is a fundamental need and vital aspect for improvement of human's quality of life [**58**]. Approximately 60 years ago, WHO described health as a mood of complete physical, psychic and social welfare rather than just not being sick [**57**,**58**]. A decade later, Jahooda criticized not being sick as psychic health criterion and instead offered multiple criteria for determining psychic health [**28**]. Unfortunately, no considerable development was observed in application of these views in scientific and practical areas for some times. In some places of the world, indices of health status has been focused yet on disease and negative concepts and fundamental concepts of Epidemiology deal with death rate but not positive performance level of people [**42**]. Health is a multidimensional concept and includes merriness and welfare feeling in addition to not being sick and unable [**32**]. Most of psychiatrists, psychologists and researches of psychic health ignore positive aspects of health [**42**,**48**]. Some efforts made to transit from traditional patterns of health have provided necessary ground for considering health as a mood of welfare (not just being sick) but are not enough. Of course new patterns of health emphasize generally on negative features and in them measuring tools of health often deal with bodily problems (mobility, ache, marital problems, etc.), psychic problems (depression, anxiety and concern) and social issues (disability in playing social role, marital problems and etc.) [**43**]. Psychic health has relation with inner enabling features or inner resources of power. Presence of these inner resources increases ability of a person for adaptive growth in spite of adverse conditions and negative events to keep his own psychic health. Psychologists and psychiatrists know a person having healthy psyche who has balance between his behaviors and control in dealing with social problems [**11**]. But study around personal and social effective determining factors on psychic health in all aspects has become a necessity for health care system of societies. A group of researchers in psychic health area has chosen a different theoretical and research approach to explain and study of this concept inspired by Positive psychology perspective. They have treated psychic health as equivalent to positive function of psychology and conceptualized it in mental health term. This group knows lack of disease is not enough for feeling health. It believes having satisfaction of life, sufficient progress, effective and efficient interaction with world, positive energy and creation of connection, desired relation with all people, society and positive progress is among features of healthy person and psychic welfare [**41**,**29**]. This has changed a study around effective personal and social determining factors on physical health and psychic welfare into a necessity for health care system of societies. Vising and Vanaden recognized a general mental health factor in 1997 and described it as a combination of special qualities including solidarity feeling, life satisfaction, emotion balance and general approach to optimism or positive orientation to life [**15**]. 

Reef knows mental health as person’s effort for vindicating his real potential abilities. Reef's model has been formed and developed through merging different theories of personal growth (like Maslow’s self-actualization and Rajerz’s perfect person) and adaptive performance (like Jahooda’s positive psychic health theory) [**17**]. Based on exact review of research literature and developmental theories solidarity Reef reminded that these views include similar and supplementary criteria of positive mental health [**43**]. Mental health is a multi dimensional context that includes merriness and hope in addition to being healthy and lack of disability [**32**,**54**]. Theoretical dimensions of positive mental health in recent view involve independency, environmental dominance, personal maturity, being helpful in living, concrete relationships with others, and self-approval. Mentioned pattern has been studied vastly in all over the world [**3**,**18**,**42**]. In the other words, mental health is linked to personal and environmental factors and healthy life condition as well as healthy body. How environment and other factors influence human’s mental health structure and with which way they deal with environmental conflicts are matters proposed in different psychological approaches and each describe human’s mental health in a special way according to their own views about human nature and his impetus. Meanwhile two factors considered in describing individuals’ mental health by researches are self-controlling and parents’ kid parenting technique. Investigations suggest that self-controlling has positive relation with psychic and physical health and reduces negative effects of stress as an inner resistance source and prevents from happening of psychic and physical disorders and generally lead to rise of person’s welfare [**8**,**30**]. In a condition that injured people’s psychic welfare is influenced by negative consequences of undesirable events, self-controlling and self-toleration are personal inner sources that can moderate stress and disability levels in undesirable conditions and cause less brilliance of negative effects of stress [**5**]. One of important skills which are characteristic of individuals’ psychic welfare is that they have self-controlling. People who can prioritize realistic purposes and make balance between feelings and wisdom while making decision are self-controlling [**4**,**6**]. Self-controlling represents adaption rate of self-behavioral characteristics and available conditions and situation [**31**]. Self-controlling concept which was developed by Schneider means a how flexible or stable is a person in a special situation [**30**]. Results of studies show people with high self-controlling have highest social skill and desirable mental health [**19**]. Log describes self-controlling as this: addressing those behaviors which results in further delayed rewards. Self-controlling can be seen in various aspects for example as a delay in gratification and practically as time duration that a person waits to reach more valuable but later consequence [**33**]. Adolescents use self- controlling skills when they want to reach a long term purpose. For this they must waive from enjoyment of food, alcohol, gamble, spending money, etc. They did this through controlling temptations of lying, escape from made promise and also calming themselves for obtained failure. In many complicated and dual situation which need having choice by the adolescent, he must use self-controlling. So deficiency in self-controlling is main core of many problems in front of the adolescent. Insufficiency in self-controlling has relation with impulsiveness and anxiety and is indicative of inability in thinking about consequences of the behavior. Therefore, recognition and determination of influence of this important variable seem necessary on mental health. On the other hand, parents’ kid parenting technique has meaningful influence on formation of children’s thoughts, behavior and emotions. According to vulnerability-stress model in psychic pathology, a number of researches have investigated role of factors related to the family as underlying factor in person’s vulnerability and psychic welfare [**25**,**26**]. Family has been always considered by related specialists due to its certain influence on children’s social and psychic growth. It is basis of community formation and keeping human emotions as smallest social unit and every deficiency in family's performance causes undesirable effects on normalizing children [**45**]. Most of incompatible and problematic people live in families having inconsiderate and autocratic style and children in families with severe conflicts show further incompatible behaviors due to lack of psychic peace, further lack of concentration and disturbance and suffer from weaker mental health [**2**,**59**]. 

In other words, relation between parents and children and other members of family could be observed as a system or network that interacts with per another. This system influences on children directly or indirectly through different styles and methods of child parenting. Kid parenting techniques are a set of trends, actions, and nonverbal expression that determine interaction nature of children and parents in all various situations. They have effect on different growth aspects of child of which can mention responsibility and self-controlling [**52**,**53**]. Bamyrand proposed for the first time autocratic, authoritative and inconsiderate kid parenting techniques. Based on Bamyrand’s theory, kid parenting techniques serve as medium between parents’ normal variables and children’s sociability. They also have supportive and non-supportive roles and consequences of applying each one is different on child’s evolution. Obtained results of some works have shown relation between kid parenting techniques and children’s social competencies and mental health [**10**,**60**]. Also some studies emphasize on presence of relation between individuals’ psychic health status and their kid parenting techniques. In a study Shake studied influence of parents’ child parenting methods on adolescents’ psychic health. He concluded that parents’ child parenting features have correlation with adolescents’ psychic health. For example, autocratic parents provide children with anxiety more than other parents and are in second rank after style of indifferent (inconsiderate) parents [**49**]. 

Already some researches were conducted about child parenting methods and their psychological results including role of mothers’ severe disciplinary methods in increasing children’s negative excitements [**23**], deficiency of participation, intimacy and reward in child-parents relationship as predicator of problems happening in the future [**7**], inconsiderate parents’ role in impulsiveness and aggression, dependency and responsibility in children [**9**], influence of excessive support of parents or their rejection in inner disorders of children and adolescents [**14**], autocratic child parenting effect on early identity and inconsiderate styles in identity disturbance [**24**]. Therefore, according to studies and importance of role related to self-controlling and kid parenting technique variables in explaining compatibility and psychic health, fundamental issue at present study is this: whether are self-managing and kid parenting techniques able to predict students' mental health?

## Method 

Present study is sectional-descriptive type with correlation design that was carried out with objective of investigating relationship among self-managing and kid parenting behaviors and mental health of high school juniors in Abadeh City. Main variables studied in the research include self-controlling, kid parenting method, and mental health. Statistical population of present study consisted of all students of public high schools in Abadeh in 2013-14. Sample size was obtained 370 persons according to purposes and type of the research and previous researches in this field and considering suppositions as significance level 95% and error 0.05 and chosen in stage-group sampling approach of mentioned centers. In this work, Tangney’s revised scale of self- controlling, Bamyrand’s kid parenting rate and Reef’s mental health questionnaire were used related to aims of the study: 

**A) Tangney’s self-controlling scale:** this test has been prepared by professor Tangney et al. in 2004 and has 36 items which have been provided with inspiration of previous studies and for eliminating defects of those questionnaires developed for self-controlling. Questions of this scale was answered based on Likert’s 5-point spectrum i.e. 1= it has similarity at all, 2= it has a few similarity, 3= incomparable similarity, 4= high similarity, 5= very high similarity. Total score of people partake in the test will be 36 ones in least case and 180 ones in maximum mood. 

Terms 1 to 34 are scored reversely. Tangney reported 0.89 as validity and credibility of these tools using Crohnbach’s alpha [**38**]. Spencer (2005) reported its inner correlation coefficient as 0.92 using Crohnbach’s alpha and Piquero et al. (2002) reported it as 0.84 [**39**,**40**]. Allahverdipoor et al. (2006) obtained 0.80 for its Crohnbach’s alpha in their research on high school students of Tehran City which shows high inner consistency of the test [**1**]. 

**B) Bamyrand’s kid parenting techniques questionnaire:** this questionnaire is a self-reporting tools that has been developed by Diana Bamyrand (1967) and measured 3 kid parenting techniques with 30 items and based on Likert’s 5-point scale. 3 child parenting and their related locutions include: 

**1.** Inconsiderate style: 1,6,10,13,14,17,19,21,24,28

**2.** Autocratic style: 2,3,7,9,12,16,18,25,26,29

**3.** Authoritative style: 4,5,8,11,15,20,22,23,27,30

The style with higher score is considered as dominant kid parenting technique. Credibility of this questionnaire was reported 0.81, 0.85, and 0.92 by Bamyrand in retest method for inconsiderate, autocratic, and authoritative kid parenting techniques respectively. She also reported about reliability of the survey and showed that autocracy of mother has relation with her inconsideration (- 0.50) and rational authority (- 0.52) [**21**]. Buri (1991) has reported credibility of the questionnaire among mothers and fathers groups using retest method as following: 0.81, 0,86, 0,78 were obtained for respectively inconsiderate, autocratic and authoritative methods in group of mothers and 0.77, 0.80 and 0.92 related to these methods in group of fathers. He observed autocracy of mother has reverse relation with inconsideration (- 0.38) and rational authority ( - 0.48) [**16**]. In a research done by Raisi (2004) in Iran, test reliability was reported for inconsiderate, autocratic, and authoritative styles in retest method as respectively 0.69, 0.77, and 0.73, which show acceptable credibility and validity of these tools [**40**]. 

**C) Reef’s mental health scale:** this tool is a self-reporting scale that was developed by Reef in 1989 for measuring Mental health structures. This questionnaire has 84 items and 6 subscales which has 14 locutions and is responded based on Likert’s 6- point spectrum (1= completely disagree – 6= completely agree). To obtain score of each scale it is enough to sum scores of all locutions related to considered subscale. Total score of mental health is revealed by score sum of 84 locutions. In the scale, higher score shows better mental health [**42**,**43**]. Validity and reliability of mental health scales have been reported proper in various researches. Dierendonck (2005) reported that inner consistency of all subscales were appropriate and their Crohnbach's alpha was between 0.77- 0.90 [**20**]. Schmitt and Reef (1997) obtained inner consistency of mental health between 0.82- 0.90 [**47**]. In another work, Reef found inner consistency coefficient of scales between 0.86- 0.93 [**42**]. Bayani et al. (2002) reported credibility and confirmation of the survey between 0.89- 0.90 in Iran showing desirability of this tool in Iranian sample [**11**]. Finally, data was analyzed which was gathered by statistical software SPSS and there is possibility of performing descriptive and inferential statistics. However, main methods for analysis of assumptions in this plan include: frequency, mean, percent, and also tests of Pearsonian correlation coefficient and multivariable regression analysis were used simultaneously to determine relation between studied variables. 

**Research questions**

1. How much is predicting validity of students’ mental health based on self-controlling? 

2. How much is predicting validity of students’ mental health based on kid parenting techniques? 

3. How is comparison of predicting validity of students’ mental health based on self-controlling and kid parenting techniques? 

**Findings**

**4-1 descriptive information related to sample group **

In this part, interpretive and presumed statistics and information have been offered. **Table 1** presents average and nominal deviation of variables studied in the research with understudied sample. 

**Table 1:** average and nominal deviation

scale	variable	mean	Standard deviation
Psychological welfare	Self-acceptance	47.10	6.257
	Positive link with others	45.25	6.315
	autonomy	46.18	5.59
	Dominance on environment	46.91	6.05
	Purposeful life	46.27	5.58
	personal growth	44.99	6.77
	Total score	276.72	13.87
Self-controlling	Self-controlling	97.52	13.50
Child parenting	Inconsiderate style	28.46	4.71
	Autocratic style	29.32	3.65
	Authoritative style	28.78	4.12

As findings of **Table 1** show highest and lowest score means was respectively dedicated to self-acceptance scale (M=47.10, SD=6.257) and personal growth scale (M=44.99, SD=6.77) in mental health survey. Average and nominal deviation of self-controlling were 97.52 and 13.50 respectively. Highest mean score in questionnaire of kid parenting technique was obtained in scale of autocratic kid parenting technique (SD=3.65, M=29.32). **Table 2** shows correlation matrix of self-controlling, kid parenting methods and mental health areas.

**4-2 correlation between under studied variables**

**Table 2:** correlation matrix between self-controlling, kid parenting techniques and mental health areas.

	Psychological Welfare	Self -acceptance	Positive relationship with others	Autonomy	Dominance on environment	Purposeful life	Intellectual development	Inconsiderately	Autocratic	Authoritatively
Self-acceptance	0.30**	1.00								
Positive link via others	0.22**	0.04	1.00							
Autonomy	0.47**	-0.13	-0.19	1.00						
Dominance on environment	0.36**	0.14*	-0.24**	0.05	1.00					
Purposeful life	0.44**	-0.31**	-0.03	0.21**	0.04	1.00				
Personal growth	0.49**	-0.10	**-0.123	0.23**	-0.14*	0.20**	1.00			
Inconsiderate style	0.01	0.04	-0.07	-0.15**	0.25**	-0.02	-0.02	1.00		
Autocratic style	-0.12*	-0.23**	-0.08	-0.05	0.12*	0.114*	-0.12*	-0.22**	1.00	
Authoritative style	0.13*	0.11**	-0.09	0.10*	0.02	0.07	0.10	-0.09	-0.06	1.00
Self-controlling	-0.16**	-0.25**	0.05	-0.08	-0.20**	0.06	0.05	0.09	-0.04	0.01
*:p=<0.05 **:p=<0.001										

Findings of **Table 2** show that correlation between under studied variables has different levels and directions in this research. Highest score of direct relationship can be seen in the element of personal growth (r=0.49) and its lowest score can occurred in the element of direct relationship via others (r=0.22) with total score of mental health rate. Generally, element of direct relationship with others has lowest correlation comparing with other Elements and scales in the study. 

Among parts of mental health rate and kid parenting method, highest rate of positive correlation was observed in element of dominance on condition (r=0.25) with inconsiderate kid parenting technique while highest rate of negative correlation was seen in element of self-acceptance (r=0.23) with autocratic kid parenting technique. Also 2 parts of self-acceptance (r=0.25) and dominance on condition (r=0.20) had highest negative correlation coefficient with self-controlling scale. 

Among different styles of child parenting, only two styles of inconsiderate and autocratic child parenting had negative relationship factor (r=0.22) with per another. There has not been observed meaningful correlation between aggrasive method and two other styles (**Table 2**). 

**4-3 investigating predictability of self-controlling on mental health**

To calculate predictability of self-controlling on mental health and its elements, linear regression approach utilized via technique of enter of all variables simultaneously. Then tables of regression score of self-controlling scale on total score obtained from mental health scale and a table including its all elements were investigated. Results of calculating regression for self-controlling predictability regarding mental health present presence of importance statistical relation (r=0.158, r2= 0.025, F=8.94, P=0.003). Therefore, investigation of predicting potential and direction of self-controlling about mental health was considered in next step. **Table 3** presents findings of the study. 

**Table 3:** Results of calculating regression predictability of self-controlling about mental health

Predicting variable	P	T	β	B
Self-controlling	0.003	2.99	-0.158	-0.164

Through investigating results represented in **Tables 3**,**4** and according to β value obtained in mental health variable, high and reverse predicting potential of self-controlling scale was considered (β=- 0.158, t=2.99, P=0.003). Consequently, research supposition is supported and statistical supposition is rejected. So, according to results we could report that in the research, the higher is person’s self-controlling, the lower success can be predicted for person in providing conditions that lead to mental health. Or in other words, self-controlling acts as an inhibiting factor in learning necessary abilities for mental health. 

**4-3-1 investigating predictability of self-managing about elements of mental health scale**

To determine predictability rate of self-controlling about six elements of mental health scale, linear regression method was used with technique of enter of all variables simultaneously. Calculation results of the regression have been brought in two **Tables 4** and **5**.

**Table 4:** Primary results of measuring variance of self-controlling variable in different aspects of mental health

mental health aspects	R	R2	F value	Significance level
Self-acceptance	0.252	0.064	23.74	0.0001
Positive relationship with others	0.048	0.002	0.821	0.336
Autonomy	0.081	0.007	2.291	0.131
Dominance on environment	0.200	0.040	14.94	0.0001
Purposeful life	0.062	0.004	1.35	0.264
Personal growth	0.054	0.003	1.037	0.309

As the results in **Table 4** show self-controlling variable has predicting potential for two elements of self-acceptance (r=0.252, r2=0.064, F=23.74, P=0.0001) and dominance of environment (r=0.200, r2=0.040, F=14.94, P=0.0001). Next investigations were done with these conditions. **Tables 4**,**5** show results investigation of ability and direction of this scale for 6 aspects of mental health scale. 

**Table 5:** Self-controlling regressions regarding mental health elements

	Predicting variable: self-controlling	B	β	T	P
Mental health aspects	Self- acceptance	-0.118	-0.252	4.87	0.0001
	Positive relationship with others	0.26	0.062	1.161	0.246
	Autonomy	-0.033	-0.081	1.514	0.131
	Dominance on environment	0.089	-0.200	3.807	0.0001
	Purposeful life	0.062	0.062	1.161	0.264
	Personal growth	0.027	0.054	1.018	0.309

According to obtained data that has been brought in **Tables 4**,**5**, self-managing parameter has high and opposite predicting ability in 2 parts of mental health i.e. self-acceptance (β=- 0.181, t=4.87, P=0.0001) and dominance on environment (β=- 0.200, t=3.807, P=0.0001). Therefore, research supposition is supported in these elements and is rejected in other elements. In other words, the results suggest that self-acceptance and person’s dominance on environment will decrease wherever person’s self-controlling is higher. It means self-controlling variable can influence as inhibiting variable on accepting abilities and weaknesses and also providing proper self-confidence in ability of dominance on condition.

**4-4 Investigating predictability of kid parenting technique about mental health**

To calculate predictability of self-controlling about mental health and its elements, linear regression method was used with technique of enter of all variables simultaneously. Then tables of regression score of self- controlling scale on total score obtained from mental health scale and a table including its all elements were investigated. Results of calculating regression for predictability of kid parenting techniques about mental health represent presence of significance statistical relation (r=0.177, r2= 0.031, F=3.85, P=0.01). These results justify presence of at least a significance statistical relation between various child-parenting styles. Therefore, investigation of predicting potential and direction of kid parenting techniques about mental health was considered in next step. **Table 6** presents findings of the study.

**Table 6:** Calculation results of predictability regression of child parenting about mental health

Predicting variable	B	β	T	P
Inconsiderate style	-0.011	-0.004	0.068	0.946
Autocratic style	-0.428	-0.113	2.12	0.035
Authoritative style	0.434	0.130	2.437	0.014

Through investigating results represented in **Table 6** and according to obtained β value, high predicting potential in 2 distinct directions in two autocratic (β=- 0.113, t=2.12, P=0.035) and authoritative (with direct prediction β=0.434, t=2.437, P=0.014) styles was observed. Consequently, research supposition is supported in these two styles and statistical supposition is rejected. So, according to findings we can say that in the study, desirable mental health can be reached by applying authoritative kid parenting technique and avoiding autocratic style. In other words, results of the current study propose that inconsiderate kid parenting technique has no influence on people's mental health. So, zero supposition is supported for this style. 

**4-4-1 Investigating predictability of kid parenting technique about elements of mental health scale**

To determine predictability rate of three child-parenting styles about 6 elements of mental health scale, linear multivariable regression method was used with technique of enter of all variables simultaneously. Calculation results of these regressions have been brought in two **Tables 7** and **8**. 

**Table 7:** Preliminary results of variance for child parenting variable in different aspects of mental health

Mental health aspects	R	R2	F value	Significance level
Self-acceptance	0.264	0.070	8.89	0.0001
Positive relationship with others	0.160	0.025	3.104	0.027
Autonomy	0.185	0.034	4.197	0.0001
Dominance on environment	0.319	0.102	13.427	0.0001
Purposeful life	0.062	0.004	1.35	0.264
Personal growth	0.139	0.019	2.353	0.070

According to results brought in **Table 7** it can be observed that kid parenting technique do not have enough variance for proper comparison of groups only in two elements of purposeful life (r=0.022, r2=0.004, F=1.35, P=0.264) and personal growth (r=0.139, r2=0.019, F=2.353, P=0.07). In this condition, it is expected that at least one of the styles has high ability for prediction in other views of mental health. Accordingly, next investigations conducted. **Table 8** shows investigation results of ability and direction of this rate for 6 aspects of mental health scale. 

**Table 8:** Calculation results of predictability regression for child parenting about mental health

mental health aspects	Predicting variable	P	T	β	B
Self-acceptance	Inconsiderate style	0.962	0.047	0.002	0.003
	Autocratic style	0.000	4.542	-0.239	-0.393
	Authoritative style	0.056	1.920	0.099	0.145
Positive relationship with others	Inconsiderate style	0.050	-1.967	-0.106	-0.144
	Autocratic style	0.041	-2.049	-0.110	-0.190
	Authoritative style	0.053	-1.944	-0.102	-0.157
autonomy	Inconsiderate style	0.004	-2.874	-0.154	-0.186
	Autocratic style	0.164	-1.394	-0.075	-0.114
	Authoritative style	0.119	1.563	0.082	0.111
Dominance on environment	Inconsiderate style	0.000	5.794	0.300	0.356
	Autocratic style	0.000	3.802	0.196	0.296
	Authoritative style	0.208	1.260	0.064	0.085
Purposeful life	Inconsiderate style	0.850	0.189	0.010	0.012
	Autocratic style	0.025	2.253	0.121	0.185
	Authoritative style	0.131	1.513	0.080	0.108
Personal growth	Inconsiderate style	0.488	-0.694	-0.037	-0.053
	Autocratic style	0.028	-2.199	-0.118	-0.211
	Authoritative style	0.090	1.701	0.090	0.142

With a glance at **Table 8** it can be found that autocratic style has meaningful and reverse relation in most of mental health aspects (self-acceptance β=- 0.393, t=4.542, P=0.0001; direct link via others β=- 0.144, t= 2.049, P=0.041; personal growth β=-0.028, t=2.199, P=0.028) but this relation is positive and meaningful in aspect of dominance on environment (β=0.296, t=3.802, P=0.0001). In the other hand, further applying of autocratic style leads to fall of self-acceptance level, having direct link via others, personal advance and morale increase of dominance on environment. Inconsiderate kid parenting technique has second highest predicting ability following autocratic style. This style has reverse relation with two views of direct link via others (β=0.144, t=1.976, P=0.05) and self-autonomy (β= 0.186, t= 2.874, P=0.00) and direct relation with dominance on environment (β=0. 356, t=5.794, P=0.0001). This result suggests that inconsiderate kid parenting technique reduces expecting direct link via others and ability of self-autonomy and increases tendency for dominance on environment. On other words, psychological aspect of link via others is the only aspect that has shown reverse and meaningful predicting ability in every 3 kid parenting techniques. 

**4-5 Comparison of predictability for kid parenting techniques and self-controlling about mental health**

To compare predictability of kid parenting technique and self-controlling about mental health, linear multivariable regression was used with technique of stepwise enter. In the following, first investigation of score correlation tables for child parenting and self-controlling scales about total score obtained from mental health scale and then regression tables were performed. **Table 9** includes information about correlation investigation of kid parenting techniques and self- controlling about mental health.

**Table 9:** Correlation coefficients of self-managing and kid parenting technique variables about mental health

Predicting variable	Number	R	Sig
Self-controlling	341	-0.12	0.013
Inconsiderate style	341	0.015	0.390
Autocratic style	341	-0.126	0.010
Authoritative style	341	0.143	0.004

As findings obtained from **Table 9** show, primary results of investigating correlation of self-managing and kid parenting techniques via mental health suggest vital link of three variables of self-controlling, autocratic, and authoritative styles with mental health. The variable “inconsiderate style” was excluded from calculation process because of its lower correlation rate with mental health. Therefore, next steps of regression were performed based on three other variables. 

**Table 10:** Predictable variance amount as step wise by under studied variables in predicting mental health

Predicting variable of variance	R	R2	F value	Significance level
Authoritative style	0.143	0.021	7.109	0.008
Self-controlling and authoritative style	0.188	0.035	6.168	0.002
Self-controlling and authoritative and autocratic styles	0.225	0.051	5.987	0.001

Based on results represented in **Table 10**, variable of authoritative style has alone predicting ability 0.143 of mental health variance variable. By adding two other parameters i.e. self-managing and aggressive method, this figure increases to 0.188 and 0.225. Accordingly, regression coefficients of these variables were calculated in stepwise way. **Table 11** presents these results. 

**Table 11:** Different models of regression with stepwise increase of variables for calculating predictability of variables having ability to predict mental health

Stage	Variables	P	T	β	B
One variable	Authoritative style	0.008	2.666	0.143	0.505
Two variables	Authoritative style	0.008	2.684	0.143	0.506
	Self-controlling	0.024	2.267	-0.121	-0.140
Three variables	Authoritative style	0.010	2.599	0.138	0.487
	Self- controlling	0.019	2.365	-0.126	-0.145
	Autocratic style	0.02	2.337	-0.124	-0.427

With reference to results obtained from **Table 11**, it is understood that highest rate of predicting potential belongs directly to aggressive method of kid parenting and following that is reversely in self-managing and autocratic method. 

## Discussion 

Before starting discussion about findings and results, it is valuable to mention that demographic information of testees was offered based on gender, education grade, field of study, education stand of parents and economic condition of the family and testees were matched based on this information. However, 56.5% and 43.5% of participants were among girls and boys respectively. Most of studied sample students (48.6%) have educated in second grade in high school. Also most of them have studied in experimental field. About education stand of parents, most of fathers (50.3%) had diploma and most of mothers (47.6%) were with education stand of under diploma. In addition, most of studied sample (67.4%) reported their economic status in medium level. Average score of students’ self-controlling was reported 97.52% and this figure suggests desirable status of self-controlling in the sample of present research. Highest score in elements of mental health scale was dedicated to self-acceptance with 47.10%, which shows the students have more desirable status in self-acceptance. Also, autocratic kid parenting technique has received highest average (29.32%) among elements of kid parenting technique. This finding implies that autocratic kid parenting technique has been used more than other styles by parents of the students. 

The findings represent correlation between studied variables has different levels and directions in this research such that highest rate of positive correlation for total score of mental health scale can be observed with personal growth element and its lowest rate is with direct link via others. Generally, element of positive relations with others has lowest correlation with other elements and scales of this work. Among parts of mental health scale and kid parenting technique, highest positive correlation was observed between element of dominance on condition and inconsiderate kid parenting technique and highest negative correlation belonged to element of self-acceptance and autocratic kid parenting technique. Also, 2 parts of self-acceptance and dominance on condition have highest negative correlation coefficient with self-controlling scale. Among various styles of child parenting, only 2 methods of autocratic and inconsiderate child parenting have negative correlation coefficient with each other. No meaningful relation of correlation has been shown between authoritative style and two other styles. As findings about first question of the study i.e. “how much is predicting ability of students’ mental health based on self-controlling?” show results of calculating predicting regression of self-controlling about mental health, suggest presence of meaningful statistical relation. According to findings and β value obtained in mental health variable, it can be concluded that self-managing has great and opposite predicting potential regarding mental health. These findings are along with findings of other studies [**8**,**13**,**30**,**50**,**56**]. Findings of these researches significantly suggest self-controlling has high predicting potential about variants like social compatibility, responsibility, solving conflict, psychic health, and mental health. However, in most of studied, direction of predicting potential of self-controlling about these variables were positive while in present study, self-controlling has reverse predicting ability about mental health. Therefore, based on findings it could be stated that in the research, the higher person’s self-controlling, the lower success is expected for person in creating conditions that lead to mental health. In other hand, self-controlling acts as an inhibitive factor in learning necessary abilities for mental health. For predictability of self-controlling regarding mental health scale, research findings show self-controlling variable has great and opposite predicting potential in 2 parts of mental health scale i.e. self-approve and dominance on environment. In other elements, self-managing did not reveal significant statistical predicting potential. In other hand, the findings of the study indicate self-acceptance and dominance on condition rates reduce wherever person’s self-controlling is higher. That means that self-controlling can be influencing as an inhibitive variant in accepting abilities and weaknesses and also providing proper self-confidence in ability for dominance on condition. In explaining these results it could be reported that self-managing is an unique personality feature that is different in various people i.e. individuals who have high self-controlling show different reactions according to their level of self-controlling and behave differently or people with high self-controlling prefer compatibility and accordance with their environment rather than dominance on environment and self-acceptance and this area requires necessity of doing accurate and extensive studies by specialists. Also Mayer and Salovey introduce self-controlling under title of correct application of excitements and believe power of adjusting feelings cause increase of personal capacity to mitigate himself, understanding popular anxieties, depressions or impatience [**36**]. People with weak self-controlling encounter permanently with hopelessness, depression and uninteresting to action and use weaker psychological skills. While people with strong skill in this area can pass misfortunes more quickly and have desirable elements of mental health [**44**] and this finding is not in agreement with findings of present study. As findings about second question of the research i.e. “how much is students’ predicting ability of mental health based on kid parenting techniques” show results of calculating predicting regression of kid parenting techniques about mental health represent existence of significant statistical relation. These results suggest existence of at least one significant statistical relation between various styles of kid parenting. According to research findings and obtained β value, high predicting potential about mental health in 2 sides in 2 autocratic and authoritative styles can be understood. Based on this finding of the research it can be seen that utilizing authoritative kid parenting technique and avoiding autocratic style can lead to desirable mental health. Findings of the current research suggest that inconsiderate kid parenting technique has no effect of individuals’ mental health. These findings are in agreement via findings of other studies [**22**,**27**,**34**,**35**,**39**,**40**,**55**]. Findings of these studies significantly justify that there is vital link between type of kid parenting technique and psychic health and mental health. 

By reviewing previous studies, it seems that adolescents and youth are self-confident, calm, and hopeful in families with authoritative style and their personal identity is not injured. Also this method accompanies with further attachment to parents especially during childhood and further satisfaction of life and provides ground for further feeling of being worthwhile and self-confidence in adolescent and as a result more desirable psychic health status. Autocratic behave via kids can provide ground for happening of mental disorders and derangement as well as undesirable emotional effects and low self-confidence. In this regard, some experts believe authoritative kid parenting technique relates to individuality and independency feeling of the child in the family unlike autocratic techniques which is so vita in promoting psychic health and mental health. While autocratic kid parenting technique injures individuality and independency of kid in the family with cruel, limiting and extreme controlling treatments [**7**]. For predictability of kid parenting techniques regarding six parts of mental health, research findings show autocratic kid parenting technique has meaningful and reverse relation with most aspects of mental health like self-acceptance, positive relations with others and personal growth but in aspect of dominance on environment, this relation is meaningful and positive. Further applying of autocratic style leads to reduction level of self-acceptance, direct link with others and personal growth and morale increase of dominance on environment. Also these findings represent inconsiderate kid parenting technique has second rank for maximum potential of predicting following autocratic style. This style has reverse relation with two aspects of positive relations with others and autonomy and direct relation with dominance on environment. It is concluded that inconsiderate kid parenting technique reduces persons' expectation for positive relations with others and ability of autonomy and increases person’s tendency to dominate on environment. In other words, psychological aspect of relations with others is the only aspect about which each 3 kid parenting techniques have shown meaningful and reverse predicting potential. Results of present research are in agreement with results of previous researches [**7**,**27**,**55**,**40**] and show child parenting methods have meaningful influence on psychic health and mental health. In other words, autocratic kid parenting technique has worst influence on status of mental health comparing to other styles. It can be mentioned that the more autocratic style is used, the more providing conditions and factors are inhibited which lead to mental health. Also, inconsiderate method results in undesirable status in psychic health and mental health for children because of rise of autonomy chance for children and ignorance of parents about them instead of disciplinary behavior following autocratic style. Generally, findings of this research characterize child parenting methods influence status of mental health. Such that ability of predicting for each method about mental health is different and authoritative style has maximum ability of direct prediction about mental health. While autocratic style has meaningful and reverse relation with mental health. Also, autocratic and inconsiderate child parenting methods have respectively maximum meaningful and reverse ability for prediction about 6 elements of mental health. 

As findings about third question of the research i.e. “how is comparison of students’ predicting ability of mental health based on self-controlling and kid parenting techniques” show authoritative child parenting method has highest ability of direct prediction about mental health and is followed by self-managing and autocratic method with reverse but not direct prediction. In explaining these findings, it can be noted that in authoritative child parenting method, parents have high level of control and responding and their children are social and effectively competent and have less behavioral problems [**12**] and high mental health but in autocratic style, parents apply high level of control and low level of responding. They expect their children obey them and often punish their children for preventing from disobedience. In inconsiderate style, parents are so responsive unlike strict parents, permit much autonomy for children, and do not oblige them to have immature behavior [**46**]. Influence of autocratic child parenting on early identity and of inconsiderate styles on identity disorder has been supported in several researches [**24**]. Agreement is on existence of relation between kid parenting technique and different consequences including psychic pathology, behavioral problems and educational achievement [**37**,**55**]. Thompson reminded autocratic child parenting approaches as a danger for behavioral problems [**52**], Turner characterized in a research with aim of determining relation of authoritative kid parenting technique with educational achievement, self-efficacy and progress motivation on university students that authoritative child parenting has also positive effect on students’ educational achievement and inner motivation and education self-efficacy [**53**]. Generally, results of these studies show mental health and psychic health have direct meaningful correlation with authoritative child parenting method and reverse and meaningful correlation with autocratic child parenting method. Also, findings of present research show self-controlling has maximum meaningful and reverse ability to predict about mental health after authoritative child parenting method. Or in other words, self-controlling in this research acts as an inhibitive factor in providing mental health.

## Conclusion 

One of evaluation axes for health of different societies is their mental health. Undoubtedly, mental health plays important role in securing dynamism and efficiency for each society and meanwhile self-controlling and methods of child parenting are important as two factors influencing on adolescents’ mental health. According to results obtained from this research, mental health has direct correlation with authoritative kid parenting technique and high meaningful reverse correlation with autocratic style. It could be stated that in this research, desirable mental health will obtain by utilizing authoritative kid parenting technique and avoiding autocratic style. Also, autocratic kid parenting technique has opposite and meaningful relation with most views of mental health as self-acceptance, positive relations with others and personal growth but this relation is positive and meaningful in dominance on condition aspect. Further application of autocratic style leads to reduction level of self-acceptance, direct link via others and personal growth and morale increase of dominance on condition. In addition, findings of this research show self-controlling have high and reverse potential to prophesy mental health. Or in another direction, self-controlling acts as an inhibitive factor in learning necessary abilities for mental health. 

**Research suggestions**

1- Holding training and justifying programs for families and providing knowledge for parents about influence of their child parenting methods on their children’s mental health.

2- Holding courses for self-controlling skills and rate and situation of using these ability linked to students’ abilities. 

3- Attention to understanding parents’ child parenting methods and self-managing skills to promote students’ mental health.

4- Determining influencing variables on students’ mental health and trying to promote these variables such that they lead to increase their mental health and promotion of life quality. 

5- Based on great and opposite predicting potential of self-controlling variable about mental health, it is necessary to do comprehensive and exact studies about influencing mechanism of this variable on mental health by specialists and psychologists as much as feasible. 

**Research limitations**

This research has been performed on Abadeh City high school pupils of and generalization of its results to other communities must be done accurately. Also, findings of this method cannot be used generally for the student societies of other cities and provinces especially those with so different cultural, ethnic and training from Abadeh City features due to limitation of above community and it is necessary to be careful in generalizing findings of the research.

**Acknowledgement**

Authors of the paper thank education authorities of Abadeh Town, counseling unit, personnel and students of studied schools. Also they appreciate other people who assisted them in doing this study. 
